# Thymosin β_4_ and β_10_ in Sjögren’s syndrome: saliva proteomics and minor salivary glands expression

**DOI:** 10.1186/s13075-016-1134-7

**Published:** 2016-10-06

**Authors:** Silvia Bosello, Giusy Peluso, Federica Iavarone, Barbara Tolusso, Irene Messana, Gavino Faa, Massimo Castagnola, Gianfranco Ferraccioli

**Affiliations:** 1 Division of Rheumatology, Fondazione Policlinico Universitario Agostino Gemelli, Rome, Italy; 2 Institute of Chemistry and Clinical Biochemistry, Catholic University, Rome, Italy; 3 Department of Life and Environmental Sciences, University of Cagliari, Cagliari, Italy; 4 Department of Surgery, Section of Pathology, University of Cagliari, Cagliari, Italy; 5 Institute of Rheumatology and Affine Sciences, Universita’ Cattolica del Sacro Cuore, Presidio Columbus, Via Giuseppe Moscati, 31, 00168 Rome, Italy

**Keywords:** Thymosin β_4_, Thymosin β_10_, Sjögren’s syndrome, Saliva, Proteomics, Immunohistochemistry, Minor salivary glands

## Abstract

**Background:**

In the present study, we investigated whether thymosin β (Tβ) in saliva and in minor salivary glands is differentially expressed in patients with primary Sjögren’s syndrome (pSS) and patients with autoimmune diseases (systemic sclerosis [SSc], systemic lupus erythematosus [SLE], and rheumatoid arthritis [RA], with and without sicca syndrome [ss]).

**Methods:**

Saliva specimens of nine patients with pSS, seven with ss/SSc, seven with ss/SLE, seven with ss/RA, seven with SSc, seven with SLE, and seven with RA, as well as ten healthy subjects, were analyzed using a high-performance liquid chromatograph coupled with a mass spectrometer equipped with an electrospray ionization source to investigate the presence and levels of Tβ_4_, Tβ_4_ sulfoxide, and Tβ_10_. Immunostaining for Tβ_4_ and Tβ_10_ was performed on minor salivary glands of patients with pSS and ss.

**Results:**

Tβ_4_ levels were statistically higher in patients with pSS with respect to the other subgroups. Tβ_10_ was detectable in 66.7 % of patients with pSS and in 42.8 % of those with ss/SSc, while Tβ_4_ sulfoxide was detectable in 44.4 % of patients with pSS and in 42.9 % of those with ss/SSc. Tβ_10_ and Tβ_4_ sulfoxide were not detectable in patients without associated ss and in healthy control subjects. Regarding thymosin immunostaining, all patients had immunoreactivity for Tβ_10_, and a comparable distribution pattern in the four different subgroups of patients was observed. Tβ_4_ immunoreactivity was present in patients with ss/SSc and those with ss/SLE, while it was completely absent in patients with pSS and those with ss/RA.

**Conclusions:**

Our data show that higher salivary Tβ expression characterizes patients with pSS, while Tβ_4_ sulfoxide and Tβ_10_ salivary expression was selectively present in patients with sicca symptoms. Moreover, at the immunohistochemical level in patients with pSS, minor salivary glands showed a peculiar pattern characterized by immunostaining for Tβ_10_ in acinar cells in the absence of any reactivity for Tβ_4_. These findings, taken together, suggest a different role for Tβ_4_ and Tβ_10_ in patients with pSS who have ss and other autoimmune disease.

## Background

Primary Sjögren’s syndrome (pSS) is a chronic autoimmune disease characterized by lymphocytic infiltration of the exocrine glands, including salivary and lachrymal glands, leading to qualitatively altered and diminished or absent glandular secretion. pSS is also characterized by marked B-lymphocyte hyperreactivity supported by hypergammaglobulinemia associated with the increase of a variety of serum autoantibodies [1]. The characteristic involvement of salivary and lachrymal glands, clinically evidenced by dry mouth (xerostomia) and/or dry eyes (xerophthalmia), makes pSS an interesting application field for salivary proteomic analysis. Saliva and tears are easy and noninvasive biological fluids to collect and can mirror local and systemic pathological changes of the disease [2–4]. In previous studies on the salivary proteome in patients with pSS, researchers identified a number of proteins as possible pSS biomarkers. Some of these biomarkers showed significantly different levels from those of healthy subjects, whereas other biomarkers were detected exclusively in the saliva of patients with pSS [3–6]. In general, proteins of acinar origin were reduced in patients with pSS, whereas acute inflammatory phase proteins were increased in subjects with pSS, compared with those of healthy subjects.

Recently, β-thymosins (Tβs), a versatile family of small peptides with interesting intra- and extracellular activities that include cardiac protection, angiogenesis, stimulation of wound healing, and hair growth [7–16], was identified in human saliva and in tears [17, 18]. Tβs play pivotal roles in the cell cytoskeletal system, acting as G-actin-sequestering molecules, suggesting that their release by damaged cells might play a role in tissue repair, such as in damaged cornea [19]. In particular, Tβ_4_, the most abundant Tβ in human tissues [7, 8], plays an important role in suppressing the production of interleukin-8 following stimulation by tumor necrosis factor-α, acting as an antimicrobial, anti-inflammatory, and antiapoptotic factor on gingival fibroblasts [20]. Moreover, our group recently reported that Tβs are probably involved in the development of the oral cavity and its annexes [21]. Another member of the Tβ family, Tβ_10_, is usually detectable at concentrations about five- to tenfold lower than Tβ_4_ in human tissues, but it has been reported to be overexpressed in human carcinogenesis [22] and in fetal tissues, including the developing human brain [23]. Involvement of Tβ_10_ in the development of the oral cavity, and in particular tooth germ development and tooth root formation, was reported recently in a mice study [24]. In addition, our group reported strong expression of Tβ_10_ in the human salivary glands during development [21]. Tβ_4_ sulfoxide was reported to have anti-inflammatory properties [25], being overexpressed in the presence of oxidative stress, due to its potential scavenger properties [26].

Because, to the best of our knowledge, no data had previously been reported on the presence of Tβ_4_, Tβ_4_ sulfoxide, and Tβ_10_ in human saliva of patients with Sjögren’s syndrome (SS), the aim of this study was to investigate the presence and levels of salivary Tβs and the immunoreactivity of these peptides in minor salivary glands in a cohort of patients with SS and in patients with other autoimmune diseases, with and without sicca syndrome (ss), to better understand the possible role of these peptides in patients with pSS as well as patients with ss and other autoimmune diseases.

## Methods

### Patients and control subjects

All female patients were enrolled at the rheumatology clinic at the Catholic University of Rome, and the study was approved by the Catholic University Ethics Committee in Rome. After we obtained signed informed consent, we collected saliva specimens from nine patients with pSS according to previous and revised criteria for the disease [27, 28]. Patients with pSS had a mean age of 63.0 ± 11.2 years and a mean disease duration of 12.0 ± 7.1 years (Table 1).

**Table 1:** Demographic and clinical characteristics of the patients and healthy control subjects enrolled in the study

	pSS (*n* = 9)	ss/SSc (*n* = 7)	ss/SLE (*n* = 7)	ss/RA (*n* = 7)	SSc (*n* = 7)	SLE (*n* = 7)	RA (*n* = 7)	HC (*n* = 10)
Age, years, mean ± SD	55.8 ± 13	63 ± 11	51 ± 16	61 ± 8	60.2 ± 7	48. ± 10	64.1. ± 9	56.6 ± 12.5
Disease duration, years, mean ± SD	7.9 ± 4.2	12 ± 7	12 ± 9	10 ± 7	10.4 ± 8	11 ± 8	9.4 ± 8	N/A
Anti-Ro/SSA- and/or anti-La/SSB-positive, *n*	9	0	0	0	0	0	0	0
ANA-positive, *n*	6	7	7	1	7	7	2	0
RF-positive, *n*	5	0	0	6	0	0	4	0
Focus score ≥1, *n*	6	0	0	0	N/A	N/A	N/A	0
UWS <1.5 ml/15 minutes, *n*	9	7	7	7	0	0	0	0
Schirmer test result <5 mm/5 minutes, *n*	9	7	7	7	0	0	0	0
Xerostomia, *n*	9	7	7	7	0	0	0	0
Xerophthalmia, *n*	9	7	7	7	0	0	0	0

*Abbreviation: pSS* Primary Sjögren’s syndrome, *ss* Sicca syndrome, *SSc* Systemic sclerosis, *SLE* Systemic lupus erythematosus, *RA* Rheumatoid arthritis, *HC* Healthy control subjects, *UWS* Unstimulated whole saliva, *ANA* Antinuclear antibodies, *RF* Rheumatoid factor, *N/A* Not applicable

As control subjects, we considered three age-matched groups:

1. Ten healthy subjects who had no signs or symptoms of ss and who were antinuclear autoantibody-negative, anti-extractable nuclear antigen autoantibody-negative, and rheumatoid factor autoantibody-negative
2. Twenty-one patients with ss associated with systemic sclerosis (SSc) (seven patients), with systemic lupus erythematosus (SLE) (seven patients), and with rheumatoid arthritis (RA) (seven patients); all of these patients complained of xerostomia and xerophthalmia, had a Schirmer test result <5 mm in 5 minutes, and had unstimulated whole saliva flow <1.5 ml in 15 minutes [27], and none of them had anti-Ro/SSA or anti-La/SSB autoantibodies and lymphocytic infiltration of the salivary glands
3. Seven patients with SSc, seven with SLE, and seven with RA without symptoms or signs of ss

All patients were diagnosed according to the revised international classification criteria for SSc, SLE, and RA [29–31]. The disease durations were comparable in the different patient study groups (Table 1).

In patients with pSS and with ss associated with SSc, SLE, or RA, saliva specimens were also collected after 30 minutes, 60 minutes, and 24 h from 5-mg pilocarpine intake. Patients with pSS and SLE were receiving antimalarial agents and a stable dose of prednisone <7.5 mg/day. Patients with RA were treated with methotrexate 10–15 mg/week. Patients with SSc were treated with iloprost, calcium channel blockers, and acetylsalicylic acid.

### Collection and preparation of saliva samples

Whole saliva was collected with a very soft plastic aspirator between 2:00 PM and 4:00 PM to reduce variations in concentrations associated with circadian rhythms of secretion. Samples were collected at least 30 minutes after any food or beverage had been consumed and teeth had been cleaned. A total of 0.5 ml of saliva was collected from each subject. Immediately after collection, samples were placed in an ice bath, and 0.5 ml of an acidic solution (0.2 % trifluoroacetic acid [TFA]) was added at a 1:1 ratio (vol/vol). The solution was then centrifuged at 8000 × *g* for 5 minutes at 4 °C. The supernatant was removed, and the precipitate was discarded. The supernatant was immediately analyzed by high-performance liquid chromatography (HPLC) in conjunction with mass spectrometry (MS) using a spectrometer equipped with an electrospray ionization (ESI) source. HPLC-ESI-MS was performed within 30 minutes of collection of the saliva sample.

### HPLC-ESI-MS analysis of salivary proteins

The HPLC-ESI-MS apparatus used was a Surveyor HPLC instrument (Thermo Fisher Scientific, Waltham, MA, USA) connected by a T splitter to a photodiode array detector and an LCQ Deca XP Plus mass spectrometer (Thermo Fisher Scientific). The chromatography column was a Vydac C8 column (Thermo Fisher Scientific) with a 5-μm particle diameter (column dimensions of 150 mm in length × 2.1-mm inner dimension). The following solutions were used for reversed-phase chromatography: Eluent A consisted of 0.056 % (vol/vol) aqueous TFA, and eluent B consisted of 0.050 % (vol/vol) TFA in acetonitrile/water 80/20 (vol/vol). The gradient applied was linear, from 0 % to 55 % of eluent B over 40 minutes, at a flow rate of 0.30 ml/minute. The T splitter addressed a flow rate of approximately 0.20 ml/minute toward the diode array detector and a flow rate of about 0.10 ml/minute toward the ESI source. The diode array detector was set at two wavelengths: 214 nm and 276 nm. Mass spectra were collected every 3 milliseconds in the positive ion mode. The MS spray voltage was 4.50 kV, and the capillary temperature was 250 °C. All common chemicals and reagents for the HPLC-MS analysis were of analytical grade and were purchased from Merck (Darmstadt, Germany) and Baker (Mallinckrodt Baker B.V., Deventer, the Netherlands). Deconvolution of the average ESI mass spectra was automatically performed by using MagTran 1.0 software (Amgen, Thousand Oaks, CA, USA) [32]. Experimental mass values obtained from the analysis were compared with theoretical values available from the Swiss-Prot [33] and EMBL [34] databases. The relative abundance of the different salivary Tβs was determined by using the extracted-ion current (XIC) strategy. The XIC procedure for each protein was based on the extraction from the total ion current profile of three mass-to-charge (*m/z*) values selected for each protein among the most relevant, provided that these did not overlap with the *m/z* values of nearly eluting proteins. Taking into account that constant analytical conditions were used for each sample, the numerical value corresponding to the integrated XIC peak area was used for the estimations of the relative abundance of peptides/proteins and for the statistical analysis. The calculated areas are all referred to a saliva volume of 17 μl. Standards of Tβ_4_ and Tβ_10_ were purchased from Bachem (Bubendorf, Switzerland), and the standard of Tβ_4_ sulfoxide was obtained, as previously described, by oxidation of Tβ_4_ [17].

### Immunohistochemical analyses

Samples of minor salivary glands were obtained through an incisional biopsy taken (after inducing local anesthesia) at the endobuccal border of the lower lip from six female patients affected by SS, three with ss/SSc, three with ss/SLE, and three with ss/RA, as well as from three healthy control subjects. In patients with SS, minor salivary gland samples had an average focus score >1 according to the Chisholm-Mason scoring system. The focus score was calculated as the number of lymphocytic foci multiplied by 4-mm^2^ surface in at least four informative lobules, and a focus was defined as a cluster of at least 50 lymphocytes [35].

All tissue samples were fixed in 10 % formalin, routinely processed, and embedded in paraffin. Immunohistochemistry was performed on 4-μm-thick sections using a labeled streptavidin-biotin complex system (LSAB2; Dako, Glostrup, Denmark) in an autostainer (Dako Cytomation, Carpinteria, CA, USA). Briefly, paraffin sections were deparaffinized and rehydrated, and endogenous peroxidase activity was quenched by 0.3 % hydrogen peroxide in methanol (30 minutes). Heat-induced antigen retrieval was carried out by steaming unstained sections in the target retrieval solution (pH 6.1; Dako) for 30 minutes. Serial sections were then incubated with 10 % normal goat serum in PBS for 60 minutes to block nonspecific binding, followed by incubation with a polyclonal anti-Tβ_4_ antibody (Bachem-Peninsula Laboratories, San Carlos, CA, USA) and with a monoclonal anti-Tβ_10_ antibody (Bachem-Peninsula Laboratories), diluted 1:600 and 1:500, respectively, in the blocking solution. Slides were then extensively washed with PBS containing 0.01 % Triton X-100 and incubated with a secondary reagent (EnVision kit; Dako) according to the manufacturer’s instructions. Diaminobenzidine was used as a chromogen. After additional washes, color was developed using the AEC reagent (Dako), and sections were counterstained with Mayer’s hematoxylin and mounted. Sections of reactive lymph nodes with Tβ_4_- and Tβ_10_-immunoreactive histiocytes were used as a positive control. As a negative control, the same procedure was applied with minor salivary gland sections, omitting the primary antibody.

### Statistical analyses

Statistical analysis was performed with SPSS version 13.0 software (SPSS, Chicago, IL, USA). Categorical and quantitative variables were described as numbers and percentages, respectively, as well as the mean ± SD. In the eight different patient subgroups considered, the analysis was performed by considering the differences either in the frequency of every single protein or in its levels. The Mann-Whitney *U* test and Wilcoxon’s matched-pairs signed-rank test were used to compare continuous variables. Categorical variables were analyzed using the chi-square test or Fisher’s exact test, depending on sample size restrictions. A *p* value ≤0.05 was considered significant.

## Results

### Proteomic analysis of salivary Tβ_4_ and Tβ_10_

Tβ_4_ was detected in the saliva of the vast majority of patients affected by SICCA (pSS + ss), with percentages ranging from 85.7 % in the subjects with ss/SSc and those with ss/RA to 88.9 % in subjects affected by pSS and to 100 % in patients with ss/SLE. Tβ_4_ was detected in the saliva of 90 % of healthy subjects. Salivary Tβ_4_ was detected at lower percentages in patients with autoimmune diseases and without ss, ranging from 28.6 % in the SLE group to 42.8 % in patients with SSc and those with RA (Tables 2 and 3).

**Table 2:** Thymosin β levels and frequency detection in different subgroups

	Tβ_4_	Tβ_10_	Tβ_4_ sulfoxide
pSS	3.5 ± 3.1 (*n* = 8)^a^	0.2 ± 0.3 (*n* = 6)	1.6 ± 3.3 (*n* = 4)^a^
ss/SSc	0.7 ± 1.0 (*n* = 6)^b^	0.03 ± 0.05 (*n* = 3)^a,b^	0.2 ± 0.4 (*n* = 3)
ss/SLE	0.6 ± 0.4 (*n* = 7)^b^	N/A (*n* = 1)	N/A (*n* = 1)
ss/RA	1.2 ± 1.1 (*n* = 6)	N/A (*n* = 1)	N/A (*n* = 1)
SSc	0.3 ± 0.6 (*n* = 3)^b^	N/A (*n* = 0)^b^	N/A (*n* = 0)
SLE	0.1 ± 0.2 (*n* = 2)^a,b^	N/A (*n* = 0)^b^	N/A (*n* = 0)
RA	0.2 ± 0.4 (*n* = 3)	N/A (*n* = 0)^b^	N/A (*n* = 0)
HC	0.8 ± 0.6 (*n* = 9)	N/A (*n* = 0)^b^	N/A (*n* = 0)

*Abbreviations: pSS* Primary Sjögren’s syndrome, *ss* Sicca syndrome, *SSc* Systemic sclerosis, *SLE* Systemic lupus erythematosus, *RA* Rheumatoid arthritis, *HC* Healthy control subjects, *N/A* Not applicable to the comparison between levels (as continuous variable) because of too few patients with detectable thymosins in the considered group

Values are the mean ± SD protein levels in the extracted ion current area (×10^8^). Values in parentheses are the number of subjects in whom the protein was identified

^a^ *p* ≤ 0.05 vs HC either in levels of thymosin β and/or in its presence

^b^ *p* ≤ 0.05 vs pSS either in thymosin β levels and/or in its presence

**Table 3:** Immunohistochemical and salivary detection of thymosin β in different subgroups

Group	Tβ_4_ IHC	Tβ_4_ saliva	Tβ_4_ sulfoxide saliva	Tβ_10_ saliva	Tβ_10_ IHC
pSS	−	88.9 %	44.4 %	66.7 %	+++
ss/SSc	+	85.7 %	42.9 %	42.8 %	+
ss/SLE	+	100 %	14.3 %	14.3 %	+
ss/RA	−	85.7 %	14.3 %	14.3 %	+++
HC	−	90 %	0 %	0 %	−

*Abbreviations: pSS* Primary Sjögren’s syndrome, *ss* Sicca syndrome, *SSc* Systemic sclerosis, *SLE* Systemic lupus erythematosus, *RA* Rheumatoid arthritis, *HC* Healthy control subjects, *IHC* Immunohistochemical staining, − Negative staining, + Positive staining

Tβ_4_ sulfoxide was found in a lower percentage of patients affected by SICCA (pSS + ss), ranging from 14.3 % in the ss/RA and ss/SLE groups to 42.9 % in the ss/SSc patients and up to 44.4 % in the pSS group. No healthy subjects showed the presence of Tβ_4_ sulfoxide in saliva (Tables 2 and 3).

Marked differences were observed among the different subgroups of patients affected by SICCA (pSS + ss) regarding the occurrence of Tβ_10_ in saliva. The percentage of salivary Tβ_10_ ranged from 14.3 % in the ss/SLE and ss/RA groups to 42.8 % in the patients with ss/SSc and up to 66.7 % in subjects affected by pSS.

No healthy subjects showed the presence of Tβ_10_ in saliva. In patients affected by SSc, RA, and SLE but without ss, both Tβ_4_ sulfoxide and Tβ_10_ were constantly absent in saliva (Tables 2 and 3).

When quantitative analyses of the salivary levels of thymosins were carried out, the highest levels of Tβ_4_, Tβ_10_, and Tβ_4_ sulfoxide were found in patients with pSS (3.5 ± 3.1 × 10^8^, 0.2 ± 0.3 × 10^8^, and 1.6 ± 3.3 × 10^8^, respectively) (Table 2). Tβ_4_ levels in subjects with pSS (3.5 ± 3.1 × 10^8^) were significantly higher than in the HC group (0.8 ± 0.6 × 10^8^) (*p* < 0.05).

All patients with SS and all patients with ss were treated with a single oral dose of 5 mg of pilocarpine, and their thymosin levels were evaluated before and after treatment. No differences were found in Tβ_4_, Tβ_10_, and Tβ_4_ sulfoxide levels before and after the drug intake in patients with SICCA (pSS and ss).

### Histology and immunohistochemistry

The histological picture of minor salivary glands in patients affected by pSS was characterized by the presence of an inflammatory infiltrate represented mainly by lymphocytes, with a focus score >1 in all cases in which histology was performed. On the contrary, no inflammatory infiltrate was detected in minor salivary glands taken from patients in the other groups.

**Fig. 1:**
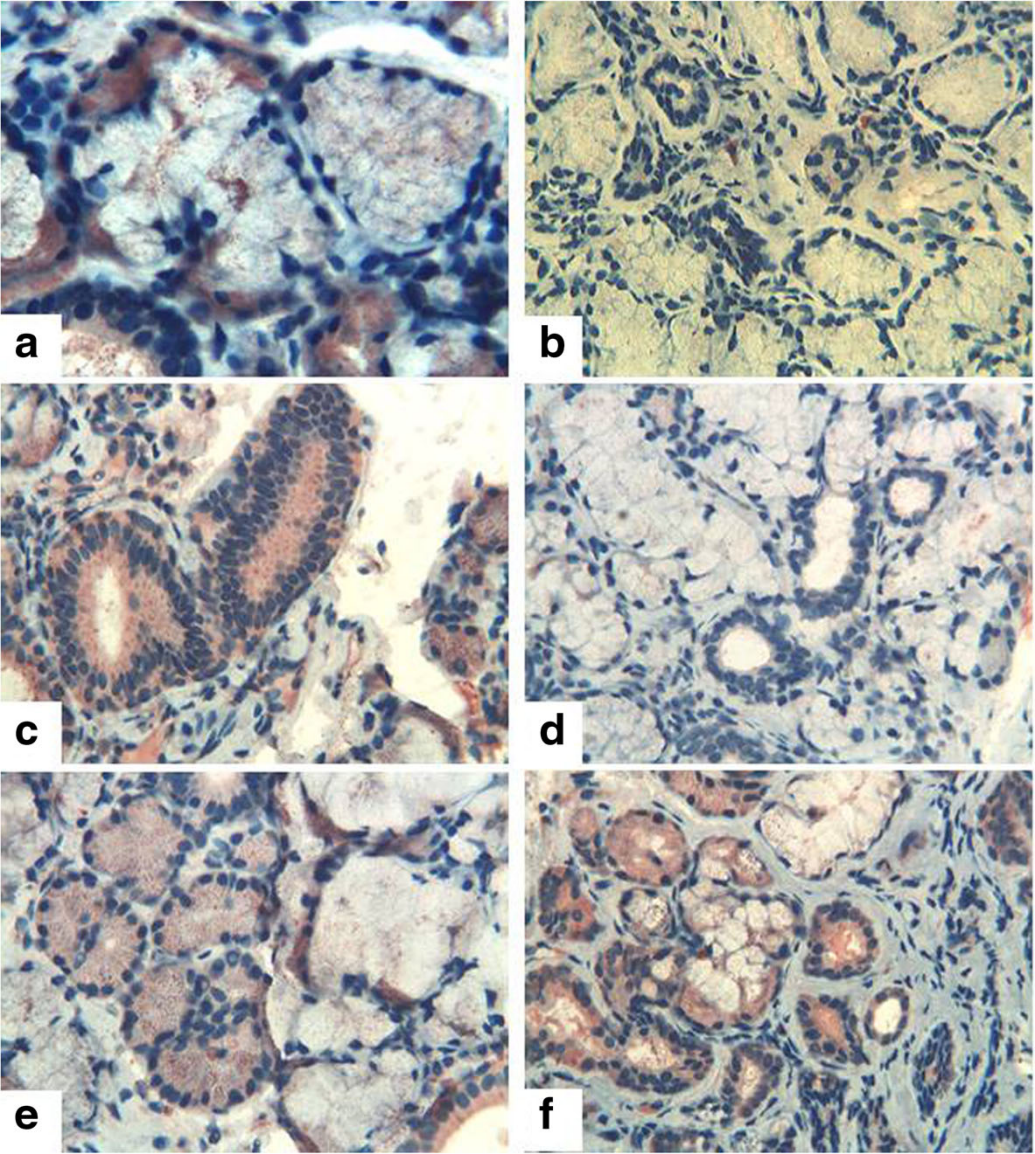
Thymosin β_4_ (Tβ_4_) and Tβ_10_ immunostaining in patients with primary Sjögren’s syndrome (pSS) and sicca symptoms and other autoimmune diseases. **a** Tβ_10_ in patients with pSS: immunoreactive granules in acinar cells. **b** Tβ_4_ in patients with pSS: No immunoreactivity is detected in acinar and ductal cells. **c** Tβ_10_ in patients with sicca syndrome and rheumatoid arthritis (ss/RA): Strong immunostaining is observed in ductal cells. **d** Tβ_4_ in patients with ss/RA: No reactivity is observed in either acinar or in ductal cells. **e** Tβ_4_ in patients with systemic sclerosis and sicca syndrome (ss/SSc): Granular immunostaining is observed in acinar cells. **f** Tβ_10_ in patients with ss/SSc: Immunoreactivity is observed mainly in serous acinar cells

At the time of immunohistochemistry, Tβ_4_ and Tβ_10_ were not detectable in the minor salivary glands of the healthy subjects. Marked differences were detected regarding the expression of Tβ_4_ and Tβ_10_ in patients affected by pSS, whereas the latter was characterized by high immunostaining, appearing as cytoplasmic granular deposits in acinar serous cells. On the contrary, immunoreactivity for Tβ_4_ was completely absent in acinar as well as ductal cells (Fig. 1). A similar immunohistochemical staining (IHC) pattern was detected in patients affected by ss/RA, with minor salivary glands being characterized by a strong, diffuse, homogeneous reactivity for Tβ_10_ in ductal cells and absence of any significant immunostaining for Tβ_4_. A different IHC pattern, characterized by immunostaining for both Tβ_4_ and Tβ_10_ in acinar cells, was observed in patients with ss/SSc or ss/SLE.

## Discussion

In SS, the proteomic analysis of saliva appears to be a very useful way to assess how the autoimmune disease affects the exocrine function of salivary glands. It is an important tool for identifying biomarkers and posttranslational modifications, as well as for identifying and quantifying peptides, proteins, and neoantigens. A number of proteins have been indicated as pSS biomarkers, showing two- to threefold up- or downregulation at significantly different levels compared with healthy subjects or having an exclusive presence in SS saliva. Proteins of acinar origin (i.e., α-amylase, carbonic anhydrase VI, proline-rich proteins, prolactin-inducible protein precursor) were reduced in patients with pSS, while inflammatory phase proteins, protease inhibitors, and antimicrobial peptides (i.e., lactoferrin, β_2_-microglobulin, immunoglobulin κ-light chain, calgranulin B, lipocalin 1 precursor, phosphatidylethanolamine binding protein, and defensins) were increased, compared with those in healthy subjects [3–6].

Recently, Tβs, which are ubiquitous peptides with interesting intra- and extracellular functions, were identified in human saliva [17, 18]. Their roles in cytoskeleton rearrangement, anti-inflammatory response, stimulation of wound healing, re-epithelialization of damaged cornea, cardiac protection, and angiogenesis [9–12] support a possible release of Tβ in the saliva of patients with oral mucosal damage, such as in pSS, and therefore a possible involvement of these peptides in the pathogenesis of pSS.

Tβ_4_ is the most abundant Tβ, representing about 70–80 % of the total Tβ content, and it is a multifunctional protein that has pleotropic activities important in cell survival and repair. It is present in all cells except red blood cells, as well as in all studied body fluid (saliva, tears, blood, plasma, wound fluid) [7–10]. Because Tβ_4_ lacks a secretion signal, it is speculated that its presence in body fluids is due to damaged cells. It is localized both in the cytoplasm and in the cell nucleus. Tβ_4_ has been shown to promote dermal and ocular repair [19], in part because of its potent chemotactic activity. It promotes the migration of various other cells, including stem cells from the hair follicles, resulting in increased hair growth [10]; tumor cells, resulting in increased metastasis; and embryonic progenitor cells from cardiac tissue, resulting in the formation of new vessels [11–13]. Tβ_4_ has been detected in human whole saliva and in tears by immunological techniques [18], and recent studies by our group evidenced that, in the oral cavity, a main contribution derives from crevicular fluid [17], where, as demonstrated by Reti and coworkers [20], Tβ_4_ plays an important role in suppressing the production of interleukin-8 following stimulation by tumor necrosis factor-α and acts on the whole saliva as an antimicrobial, anti-inflammatory, and antiapoptotic peptide on gingival fibroblasts. The ability of Tβ_4_ to block apoptosis and downregulate inflammatory cytokines may explain its anti-inflammatory activity. Tβ_4_ also increases oxidative enzymes and protects cells and tissues from oxidative damage. In different body fluids, the oxidation product of Tβ_4_ at the methionine-6 residue, Tβ_4_ sulfoxide was also detectable. Tβ_4_ sulfoxide is generated by monocytes in the presence of glucocorticoids, and, like Tβ_4_, it seems to have anti-inflammatory properties [25, 26].

Tβ_4_ frequently coexists with a second member of the Tβ family: Tβ_10_ [13]. Tβ_10_ concentration is usually about 20 times lower than Tβ_4_ in normal tissues, but the Tβ_10_/Tβ_4_ ratio seems to increase in preneoplastic and neoplastic tissues and in activated lymphocytes [13].

Whereas Tβ_4_ is a potent enhancer of angiogenesis, Tβ_10_ inhibits it, and changes in the ratio of the two peptides can exert either a positive or negative control [10]. Tβ_4_ can upregulate the expression of hepatocyte growth factor and downregulate the expression of platelet-derived growth factor-β receptor in a model of liver fibrosis, suggesting an antifibrotic potential of Tβ_4_ [36, 37].

In this study, the presence and the levels of Tβ_4_, Tβ_4_ sulfoxide, and Tβ_10_ were determined both in saliva by a proteomic approach and in salivary glands by immunohistochemistry. This integrated approach allowed us to compare Tβ_4_ and Tβ_10_ expression in saliva and in salivary glands of healthy subjects, patients with pSS, and patients with ss and other autoimmune diseases. Tβ_4_ was present in the saliva of the vast majority of patients complaining of ss symptoms and in healthy control subjects, but its levels were significantly higher in subjects affected by SS than in the other subgroups.

The high levels of Tβ_4_ found in the saliva of patients with SS contrasted with the absence of reactivity for the peptide in salivary glands observed by immunohistochemistry. This finding suggests that acinar cells of salivary glands constantly exposed to inflammatory damage of SS might release high amounts of Tβ_4_ in the saliva, where it would exert a cytoprotective effect in tissue repair and in the anti-inflammatory response, as previously shown in the damaged cornea [19]. Alternatively, the increased apoptosis of epithelial cells that characterize pSS can release the peptide, with the new and regenerating epithelial cells not producing enough peptide to be detected by IHC [38]. This is supported indirectly by the observation that Tβ_10_ is expressed more frequently than Tβ_4_ in IHC and that in fact Tβ_10_ is involved in apoptosis mechanisms [39].

Tβ_10_ and Tβ_4_ sulfoxide were selectively detectable in patients with SICCA (ss + pSS), as compared with none of the patients without associated ss or the healthy control subjects. Our data suggest that these two isoforms of Tβ could be salivary biomarkers of SICCA. The highest levels of Tβ_4_ sulfoxide and Tβ_10_ were found in patients with pSS. The increased levels of Tβ_4_ sulfoxide in pSS saliva could mirror the higher oxidative stress status of the oral cavity related to a characteristic inflammatory microenvironment due to the immunological destruction of the salivary glands typical of pSS. Indeed, the release of an increased amount of Tβ_4_ sulfoxide could represent a scavenger mechanism able to reduce the negative effects of oxidative stress on salivary proteins and enzymes, along with a cytoprotective effect, in both forms.

Tβ_10_ has a similar expression pattern of Tβ_4_ sulfoxide in pSS and ss saliva, and its presence may be due to a passive release from damaged cells, such as Tβ_4_. The increased amount of Tβ_10_ in pSS saliva could be related to the activated lymphocytes present in salivary gland infiltrates.

It has been shown that thymosins prevent scarring and fibrosis in many animal models of human pathologies through their potent regenerative and antifibrotic effect in reducing inflammation and in modulating oxidative damage. In this scenario, our data suggest a role of thymosins in pSS and in patients with ss related to other autoimmune diseases, supporting a rationale for expecting thymosins’ therapeutic effect in clinical practice in patients with ss symptoms, as well as what has already been reported in lung damage [40, 41], eye injury [19], and wound healing of the palatal mucosa [42].

## Conclusions

Proteomic analysis of the expression of Tβ_4_, Tβ_4_ sulfoxide, and Tβ_10_ mirrors the different oral microenvironment of patients with pSS and with autoimmune diseases who complain of ss. Thymosins may potentially be used as biomarkers in patients with pSS and patients with ss, and IHC analysis helped us to hypothesize their possible role. Functional studies at the epithelial cell level are necessary to further elucidate their role.

## Abbreviations

ANA: Antinuclear autoantibodies
ENA: Extractable nuclear antigen autoantibodies
ESI: Electrospray ionization
HC: Healthy control subjects
HPLC: High-performance liquid chromatography
IHC: Immunohistochemical
*m/z*: Mass-to-charge ratio
MS: Mass spectrometry
N/A: Not applicable
pSS: Primary Sjögren’s syndrome
RA: Rheumatoid arthritis
RF: Rheumatoid factor
SICCA: Primary Sjögren’s syndrome and sicca syndrome
SLE: Systemic lupus erythematosus
ss: Sicca syndrome
SS: Sjögren’s syndrome
SSc: Systemic sclerosis
TFA: Trifluoroacetic acid
Tβ: Thymosin β
UWS: Unstimulated whole saliva
XIC: Extracted-ion current

## References

[1] Fox RI (2005). Sjögren’s syndrome. Lancet.

[2] Hu S, Loo JA, Wong TS (2007). Human saliva proteome analysis. Ann N Y Acad Sci.

[3] Baldini C, Giusti L, Bazzichi L, Lucacchini A, Bombardieri S (2008). Proteomic analysis of the saliva: a clue for understanding primary from secondary Sjögren’s syndrome?. Autoimmun Rev.

[4] Fleissig Y, Deutsh O, Reichenberg E, Redlich M, Zaks B, Palmon A (2009). Different proteomic protein patterns in saliva of Sjögren’s syndrome patients. Oral Dis.

[5] Ferraccioli G, De Santis M, Peluso G, Inzitari R, Fanali C, Bosello SL (2010). Proteomic approaches to Sjögren’s syndrome: a clue to interpret the pathophysiology and organ involvement of the disease. Autoimmun Rev.

[6] Peluso G, De Santis M, Inzitari R, Fanali C, Cabras T, Messana I (2007). Proteomic study of salivary peptides and proteins in patients with Sjögren’s syndrome before and after pilocarpine treatment. Arthritis Rheum.

[7] Hannappel E (2007). β-Thymosins. Ann N Y Acad Sci.

[8] Huff T, Müller CS, Otto AM, Netzker R, Hannappel E (2001). β-Thymosins, small acidic peptides with multiple functions. Int J Biochem Cell Biol.

[9] Mannherz HG, Hannappel E (2009). The β-thymosins: intracellular and extracellular activities of a versatile actin binding protein family. Cell Motil Cytoskeleton.

[10] Philp D, Goldstein AL, Kleinman HK (2004). Thymosin β_4_ promotes angiogenesis, wound healing, and hair follicle development. Mech Ageing Dev.

[11] Bock-Marquette I, Saxena A, White MD, Dimaio JM, Srivastava D (2004). Thymosin β_4_ activates integrin-linked kinase and promotes cardiac cell migration, survival and cardiac repair. Nature.

[12] Smart N, Risebro CA, Melville AA, Moses K, Schwartz RJ, Chien KR (2007). Thymosin β_4_ induces adult epicardial progenitor mobilization and neovascularization. Nature.

[13] Hall AK (1991). Differential expression of thymosin genes in human tumors and in developing human kidney. Int J Cancer.

[14] Sun W, Kim H (2007). Neurotrophic roles of the β-thymosins in the development and regeneration of the nervous system. Ann N Y Acad Sci.

[15] Nemolato S, Cabras T, Fanari MU, Cau F, Fanni D, Gerosa C (2010). Immunoreactivity of Thymosin β_4_ in human foetal and adult genitourinary tract. Eur J Histochem.

[16] Naylor PH, McClure JE, Spangelo BL, Low TL, Goldstein AL (1984). Immunochemical studies on thymosin: radioimmunoassay of thymosin β_4_. Immunopharmacology.

[17] Inzitari R, Cabras T, Pisano E, Fanali C, Manconi B, Scarano E (2009). HPLC-ESI-MS analysis of oral human fluid reveals that gingival crevicular fluids the main source of oral thymosins β_4_ and β_10_. J Sep Sci.

[18] Badamchian M, Damavandy AA, Damavandy H, Wadhwa SD, Katz B, Goldstein AL (2007). Identification and quantification of thymosin β_4_ in human saliva and tears. Ann N Y Acad Sci.

[19] Sosne G, Chan CC, Thai K, Kennedy M, Szliter EA, Hazlett LD (2001). Thymosin β_4_ promotes corneal wound healing and modulates inflammatory mediators in vivo. Exp Eye Res.

[20] Reti R, Kwon E, Qiu P, Wheater M, Sosne G (2008). Thymosin β_4_ is cytoprotective in human gingival fibroblasts. Eur J Oral Sci.

[21] Nemolato S, Messana I, Cabras T, Manconi B, Inzitari R, Fanali C (2009). Thymosin β_4_ and β_10_ levels in pre-term newborn oral cavity and foetal salivary glands evidence a switch of secretion during foetal development. PLoS One.

[22] Santelli G, Califano D, Chiappetta G, Vento MT, Bartoli PC, Zullo F (1999). Thymosin β-10 gene overexpression is a general event in human carcinogenesis. Am J Pathol.

[23] Hall AK, Hempstead J, Morgan JI (1990). Thymosin β_10_ levels in developing human brain and its regulation by retinoic acid in the HTB-10 neuroblastoma. Brain Res Mol Brain Res.

[24] Shiotsuka M, Wada H, Kiyoshima T, Nagata K, Fujiwara H, Kihara M (2013). The expression and function of thymosin β_10_ in tooth germ development. Int J Dev Biol.

[25] Young JD, Lawrence AJ, MacLean AG, Leung BP, McInnes IB, Canas B (1995). Thymosin β_4_ sulfoxide is an anti-inflammatory agent generated by monocytes in the presence of glucocorticoids. Nat Med.

[26] De Santis M, Inzitari R, Bosello SL, Peluso G, Fanali C, Iavarone F (2011). β-Thymosins and interstitial lung disease: study of a scleroderma cohort with a one-year follow-up. Respir Res.

[27] Vitali C, Bombardieri S, Jonsson R, Moutsopoulos HM, Alexander EL, Carsons SE (2002). Classification criteria for Sjögren’s syndrome: a revised version of the European criteria proposed by the American-European Consensus Group. Ann Rheum Dis.

[28] Shiboski SC, Shiboski CH, Criswell LA, Baer AN, Challacombe S, Lanfranchi H (2012). American College of Rheumatology classification criteria for Sjögren’s syndrome: a data-driven, expert consensus approach in the Sjögren’s International Collaborative Clinical Alliance Cohort. Arthritis Care Res (Hoboken).

[29] Subcommittee for Scleroderma Criteria of the American Rheumatism Association Diagnostic and Therapeutic Criteria Committee (1980). Preliminary criteria for the classification of systemic sclerosis (scleroderma). Arthritis Rheum.

[30] Tan EM, Cohen AS, Fries JF, Masi AT, McShane DJ, Rothfield NF (1982). The 1982 revised criteria for the classification of systemic lupus erythematosus. Arthritis Rheum.

[31] Aletaha D, Neogi T, Silman AJ, Funovits J, Felson DT, Bingham CO (2010). Rheumatoid arthritis classification criteria: an American College of Rheumatology/European League Against Rheumatism collaborative initiative. Arthritis Rheum.

[32] Zhang Z, Marshall AG (1998). A universal algorithm for fast and automated charge state deconvolution of electrospray mass-to-charge ratio spectra. J Am Soc Mass Spectrom.

[33] Swiss Institute of Bioinformatics (SIB). ExPASy Bioinformatics Resource Portal [Swiss-Prot]. http://www.expasy.org/tools.

[34] European Molecular Biology Laboratory (EMBL) database. http://www.embl-heidelberg.de.

[35] Vivino FB, Gala I, Hermann GA (2002). Change in final diagnosis on second evaluation of labial minor salivary gland biopsies. J Rheumatol.

[36] Barnaeva E, Nadezhda A, Hannappel E, Sjogren MH, Rojkind M (2007). Thymosin β_4_ upregulates the expression of hepatocyte growth factor and downregulates the expression of PDGF-β receptor in human hepatic stellate cells. Ann N Y Acad Sci.

[37] Oh IS, So SS, Jahng KY, Kim HG (2002). Hepatocyte growth factor upregulates thymosin beta4 in human umbilical vein endothelial cells. Biochem Biophys Res Commun.

[38] Polihronis M, Tapinos NI, Theocharis SE, Economou A, Kittas C, Moutsopoulos HM (1998). Modes of epithelial cell death and repair in Sjögren’s syndrome (SS). Clin Exp Immunol.

[39] Hall AK (1995). Thymosin β-10 accelerates apoptosis. Cell Mol Biol Res.

[40] Conte E, Genovese T, Gili E, Esposito E, Iemmolo M, Fruciano M (2012). Protective effects of thymosin β_4_ in a mouse model of lung fibrosis. Ann N Y Acad Sci.

[41] Conte E, Fagone E, Gili E, Fruciano M, Iemmolo M, Pistorio MP, et al. Preventive and therapeutic effects of thymosin β_4_ N-terminal fragment Ac-SDKP in the bleomycin model of pulmonary fibrosis. Oncotarget. 2016;7(23):33841–54. doi:10.18632/oncotarget.8409. 10.18632/oncotarget.8409 PMC5085123 27029074

[42] Zhu T, Park HC, Son KM, Kwon JH, Park JC, Yang HC (2014). Effects of thymosin β_4_ on wound healing of rat palatal mucosa. Int J Mol Med.

